# Papillary Thyroid Carcinoma, Cushing Disease, and Adrenocortical Carcinoma in a Patient with Li-Fraumeni Syndrome

**DOI:** 10.1016/j.aace.2024.03.007

**Published:** 2024-03-28

**Authors:** Jared G. Friedman, Ioannis G. Papagiannis

**Affiliations:** Northwestern University Feinberg School of Medicine, Chicago, Illinois

**Keywords:** Li-Fraumeni syndrome, p53, adrenocortical carcinoma, endocrine neoplasia

## Abstract

**Background/Objective:**

Li-Fraumeni syndrome (LFS) is an inherited sequence variant in TP53 characterized by the early onset of various core malignancies including adrenocortical carcinoma (ACC), sarcomas, breast cancer, leukemias, and central nervous system tumors. We present a case of a patient with LFS who developed endocrine neoplasms not classically seen in LFS in addition to developing ACC.

**Case Report:**

A 26-year-old nonbinary individual assigned female at birth with a history of LFS complicated by osteosarcoma of the jaw was incidentally found to have thyroid and sellar masses on surveillance magnetic resonance imaging. Fine-needle aspiration of thyroid mass confirmed papillary thyroid carcinoma, and the patient underwent total thyroidectomy. Pituitary workup was notable for laboratory test results consistent with adrenocorticotropic hormone-dependent hypercortisolism; the patient underwent resection of the pituitary lesion. The patient was subsequently noted on abdominal imaging to have a new left adrenal mass; they underwent left adrenalectomy with pathology consistent with ACC.

**Discussion:**

There is limited literature on the relationship between LFS and thyroid and pituitary neoplasms. Genetic testing has suggested that TP53 sequence variants may play a role in tumorigenesis in thyroid and pituitary neoplasms; however, most of the current literature is based on evidence of somatic rather than germline sequence variants.

**Conclusion:**

This case highlights a patient with LFS with neoplasia of multiple endocrine organs including ACC, which is a classic finding, as well as papillary thyroid carcinoma and Cushing disease. Further investigation may be necessary to assess if patients with LFS are at a higher risk of various endocrine neoplasms in addition to the core malignancies classically described because this could affect future screening protocols.

## Introduction

Li-Fraumeni syndrome (LFS) is an inherited autosomal dominant disorder associated with a germline sequence variant in the TP53 tumor suppression gene, which results in an increased incidence of missense sequence variants and risk of subsequent malignancy.^1^ LFS was first described in 1969 in children with sarcoma and a family history of cancer and is rare and estimated to affect 1000 multigenerational families worldwide.^1^^,^^2^ The missense sequence variants in LFS are classically thought to result in 6 core categories of malignancies: soft tissue sarcoma, osteosarcoma, premenopausal breast cancer, leukemia, tumors of the central nervous system (CNS), and adrenocortical carcinoma (ACC).^3^ Malignancies are usually diagnosed before the age of 45 years.^4^ Various guidelines suggest screening protocols for the malignancies most associated with LFS.^3^^,^^5^^,^^6^ Here, we report a case of a patient with LFS with classic LFS-associated malignancies as well as nonclassic endocrine neoplasia.

## Case Report

**Fig. 1:**
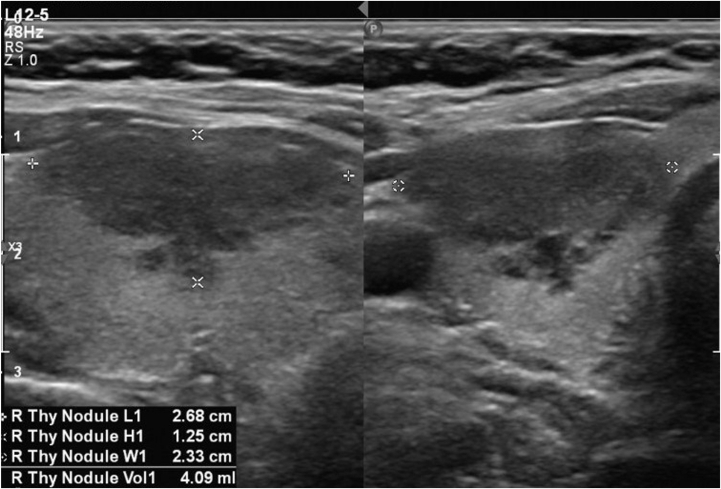
Ultrasound of thyroid demonstrating a 2.7 cm right-sided hypoechoic nodule with irregular margins.

**Fig. 2:**
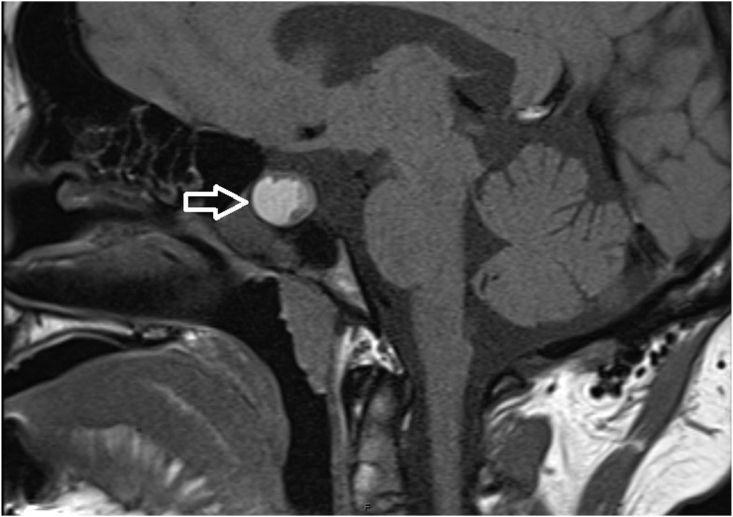
Magnetic resonance imaging of pituitary notable for a 1.4 cm well-circumscribed, hemorrhagic sellar lesion (white arrow).

A 26-year-old nonbinary individual assigned female at birth with a history of LFS (TP53 sequence variant of c818G>T) was initially referred to the Endocrinology department for abnormalities on imaging of the pituitary and thyroid glands. They were previously diagnosed with osteosarcoma of the right jaw at the age of 19 years, which was treated with surgery and chemotherapy. A body positron emission tomography/computed tomography at the time demonstrated an fluorodeoxyglucose-avid right adrenal mass; they underwent right adrenalectomy; however, the pathology revealed benign adenoma. They also obtained genetic testing at this time and were found to have a sequence variant in the TP53 gene consistent with LFS. They subsequently underwent prophylactic/gender-affirming bilateral mastectomy at the age of 25 years. The following year, magnetic resonance imaging (MRI) of the face and neck for osteosarcoma monitoring incidentally showed a new 1.7 cm right thyroid nodule and a new 2.1 cm hemorrhagic appearing sellar lesion. Follow-up thyroid ultrasound confirmed a Thyroid Imaging Reporting and Data Systems value of 4 thyroid nodule, 2.7 cm in length, with irregular margins and no suspicious lymphadenopathy (Fig. 1), and pituitary MRI showed a 1.4 cm hemorrhagic lesion in the right aspect of sella, consistent with possible hemorrhagic pituitary adenoma (Fig. 2). They denied any headaches or vision changes and were not on blood thinners. They were recommended to obtain a pituitary laboratory panel and undergo a fine-needle aspiration (FNA) biopsy of thyroid nodule, which was performed after the initial visit.

Initial pituitary laboratory tests resulted with a thyroid-stimulating hormone (TSH) level of 0.52 μIU/mL (0.40-4.00 μIU/mL), free T4 0.76 ng/dL (0.70-1.50 ng/dL), prolactin 24.5 ng/mL (2.3-26.7 ng/mL), AM cortisol 13.5 μg/dL (5.0-25.0 μg/dL), adrenocorticotropic hormone 88.4 pg/mL (7.2-63.6 pg/mL, Roche Elecsys immunoassay), and insulin-like growth factor 1 193 ng/mL (63-373 ng/mL). FNA biopsy revealed papillary thyroid carcinoma (PTC). They were referred to Endocrine Surgery where they were evaluated 2 weeks later and scheduled for surgery in the following week. They underwent a total thyroidectomy with bilateral central neck dissection. Pathology showed a 1.8 cm PTC with negative margins, no extrathyroidal extension, no vascular invasion, and 10/24 positive central lymph nodes (largest focus 0.9 cm with no extranodal extension). They started levothyroxine 175 mcg daily. Six-week postoperative testing was notable for TSH level of 0.06 μIU/mL, free T4 level of 1.35 ng/dL, serum thyroglobulin (Tg) level of 0.26 ng/mL, and negative Tg antibody. The patient did not receive adjuvant radioiodine therapy due to concerns regarding increased risk for radiation-induced secondary neoplasms in LFS.^7^

While the patient was undergoing thyroid evaluation, they concurrently underwent pituitary assessment. The initial pituitary assessment was focused on ruling out any hormonal deficiencies before thyroidectomy, given concern for hemorrhage on pituitary MRI. At the next follow-up visit 6 weeks after thyroidectomy, the patient endorsed progressive weight gain, fatigue, and easy bruising. They did not have obvious Cushingoid features on examination. Testing was notable for elevated late-night salivary cortisol levels of 0.14 and 0.22 mcg/dL (<0.09 mcg/dL) and AM cortisol level of 6.5 μg/dL (<1.8 μg/dL) after administration of 1 mg of dexamethasone with an adequate serum dexamethasone level. Urinary cortisol testing was not obtained because the complete laboratory workup was deemed sufficient for the diagnosis of endogenous hypercortisolism. Follow-up pituitary MRI showed growth of the right-sided adenoma to 1.9 cm compared with 1.4 cm on MRI 8 months before. They were referred to the Neurosurgery department who recommended inferior petrosal sinus sampling, which they underwent with confirmation of pituitary source for hypercortisolism. They then underwent endoscopic endonasal transcavernous resection of pituitary lesion notable for pituitary adenoma with Ki67 4.5%, moderate p53 staining, and weak to focally positive adrenocorticotropic hormone staining consistent with Cushing disease (CD). Postoperative day 1 AM cortisol level was 2.7 μg/dL, consistent with biochemical cure; the patient was started on replacement hydrocortisone, which was tapered off over 3 months.

**Fig. 3:**
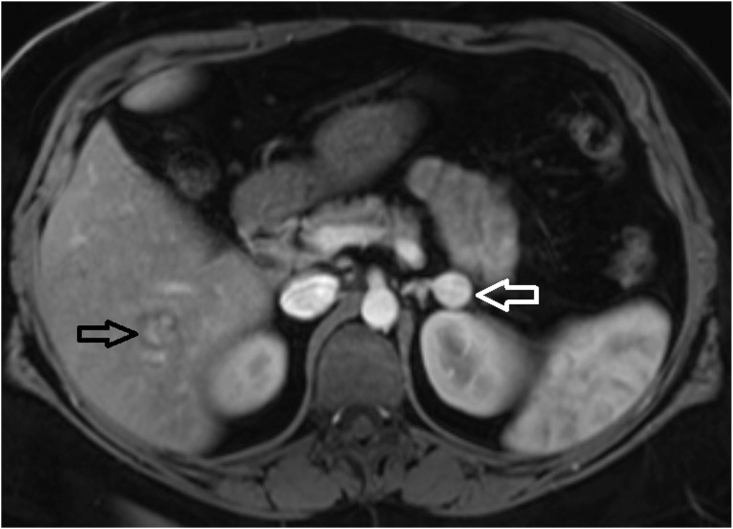
Magnetic resonance imaging of the abdomen with and without contrast showing a 2.0 cm left adrenal nodule (white arrow) and one of several hyperintense hepatic lesions (black arrow).

Approximately 4 months after surgery, they underwent a computed tomography abdomen to evaluate acute abdominal pain and were incidentally noted to have a lipid-poor 1.9 cm left adrenal nodule not seen on prior imaging. Follow-up MRI of abdomen characterized the nodule as having minimal decreased signal on opposed phase imaging; it also demonstrated 6 new hepatic lesions with the largest being 1.9 cm (Fig. 3). Plasma metanephrines and dehydroepiandrosterone sulfate were obtained and were normal. Interval MRI of abdomen with contrast 1 month later showed increase in size of left adrenal nodule to 2.5 cm and stable size of liver lesions with 2 indeterminate lesions and 4 lesions with benign imaging characteristics. The decision was made to pursue surgical resection of left adrenal gland while continuing to monitor the liver lesions with serial imaging. They underwent robotic-assisted left adrenalectomy and started replacement glucocorticoid and mineralocorticoid. Pathology revealed ACC with negative surgical margins, Ki67 20-25%, and weakly positive staining for p53. At 1 month postop, they underwent an MRI abdomen with contrast with noted growth of segment V hepatic lesion from 1.5 cm to 2.1 cm; this was one of the indeterminate lesions in the previous study based on absent contrast retention in the hepatobiliary phase. Patient subsequently underwent a core biopsy of segment V liver lesion, which appeared compatible with metastatic ACC. While awaiting management plan for presumed metastatic liver lesion, they were found on surveillance laboratory tests approximately 18 months after thyroidectomy to have an elevated serum Tg level of 1.96 ng/mL with concurrent TSH level of 0.14 μIU/mL. Neck ultrasound showed multiple heterogenous right-sided lymph nodes of >1 cm in size, which were new compared with preoperative ultrasound; FNA sampling of prominent right level III lymph node revealed recurrence of metastatic PTC. Management was delayed until the ACC treatment plan was determined.

**Fig. 4:**
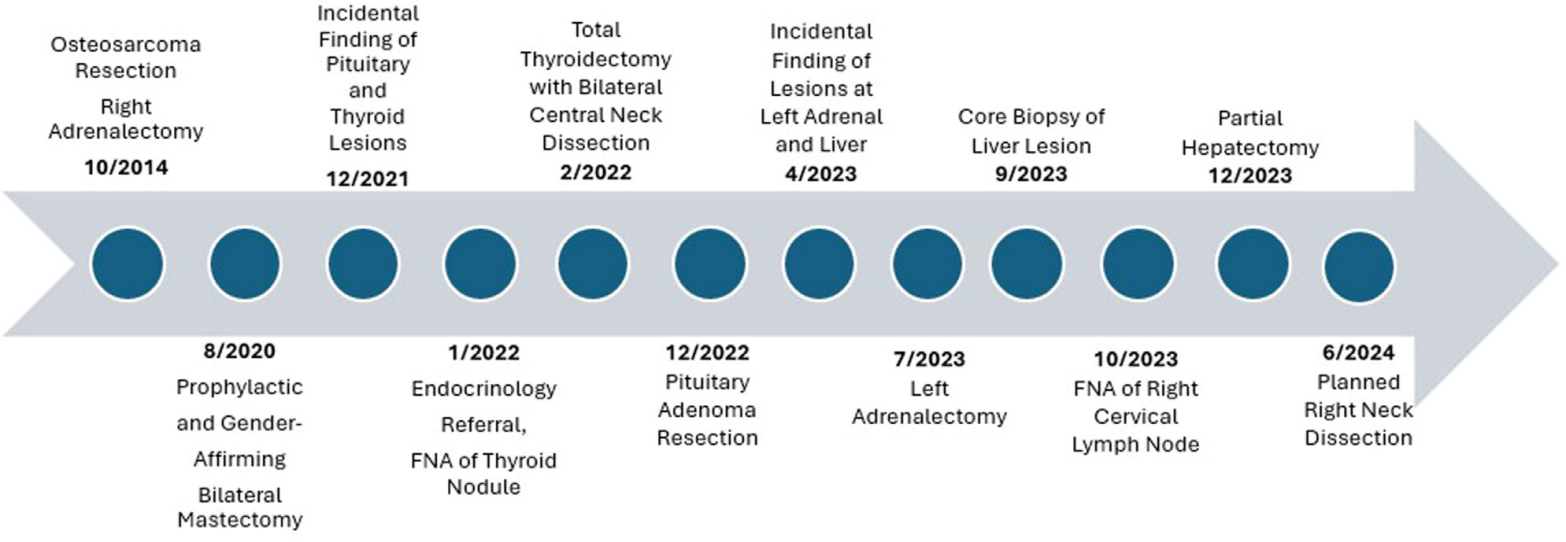
Timeline of significant clinical events beginning in October 2014 (age 19 years) through planned neck dissection in June 2024 (age 28 years).

After discussion between Endocrinology, Oncology, and Surgical teams, the patient underwent partial hepatectomy of segment V and segment II. Despite the prior biopsy results, the final surgical pathology did not show evidence of ACC and instead showed perivascular epithelioid cell tumor in segment V and focal nodular hyperplasia in segment II. Per Oncology, the perivascular epithelioid cell tumor will be monitored with serial MRIs but does not require further therapy at this time. Oncology also discussed the role of adjuvant mitotane therapy for ACC, and the patient decided to forgo mitotane, given early-stage ACC without metastases and concern for potential medication side effects. The patient has now been scheduled for right modified radical neck dissection for management of recurrent PTC. A timeline of significant clinical events can be found in Figure 4.

## Discussion

LFS is a hereditary cancer syndrome characterized by the development of multiple classic malignancies often at an early age.^1^ The most common malignancy associated with LFS in childhood and adolescence is sarcoma as in this patient who was diagnosed with osteosarcoma at the age of 19 years.^8^ The relationship between LFS and ACC is well characterized; most often, the onset is in early childhood with a median age of onset of 4.8 years for LFS-associated ACC compared with that usually after the age of 40 years for sporadic ACC.^9^^,^^10^ In 1 case registry of adult-onset ACC, TP53 was variant in only 4 out of 103 cases, but the 4 cases with TP53 sequence variant were aged <40 years.^4^

Unique to this case was the presence of 2 additional endocrine neoplasms: PTC and CD. The association of thyroid cancer and LFS has not been well characterized. The incidence of thyroid cancer in LFS has been reported in 2 cohort studies. In a Brazilian cohort of 193 individuals with LFS p.2337H sequence variant, 101 developed malignancy and 11 of them developed PTC with a mean age of diagnosis of 44 years.^11^ In contrast, a French cohort of 415 LFS carriers reported a thyroid cancer incidence of 0.9%.^12^ Acquired p53 sequence variant has been associated with anaplastic thyroid cancer but an increased risk of anaplastic thyroid cancer has not been reported.^13^ Overall, the incidence of PTC in the United States has been increasing possibly due to increased detection because now it is estimated that 1 in 55 women and 1 in 149 men will be diagnosed with thyroid cancer in their lifetime.^14^ However, with more genetic testing of thyroid samples, it has been reported that up to 40% of PTC has TP53 sequence variant, which is thought to play a role in early tumorigenesis of thyroid cancer.^15^ At present, it is unclear if PTC is a component of LFS, and thyroid ultrasound is not currently included in LFS screening protocols.^11^

Although Cushing syndrome has been described in LFS in the setting of autonomous adrenal cortisol secretion in ACC, neither CD nor pituitary neoplasia has been classically described in LFS.^16^^,^^17^ The CNS tumors of LFS are typically gliomas and astrocytomas rather than pituitary adenomas as in this patient.^3^ In 1 case, a 6-year-old boy with germline TP53 sequence variant did present with a pituitary lactotroph tumor with mass effect requiring surgery with patchy positivity for p53 on immunohistochemistry; he had recurrence requiring another surgery and radiation and later developed medulloblastoma.^18^ A meta-analysis showed that somatic TP53 sequence variant was found in 8 out of 64 cases of CD (12.5%); these tumors were typically larger and more aggressive.^19^ Another analysis of functional corticotroph tumors found a pathogenic variant of TP53 in 9 out of 86 cases, but it was thought that these were somatic rather than germline sequence variants because all patients were aged at least 30 years and lacked additional malignancies seen in germline TP53 sequence variant.^20^ Although current LFS screening protocols suggest CNS imaging to assess for tumors, there are presently no recommendations for dedicated pituitary imaging or functional testing.^5^^,^^6^

## Conclusion

LFS is a rare hereditary condition with increased risk of specific core malignancies at a young age including ACC. To our knowledge, this case appears to be the first report of adrenal, thyroid, and pituitary neoplasia in an individual with LFS and suggests that patients with LFS may be at a higher risk of various endocrine neoplasms in addition to the core malignancies classically described.

## Disclosure

Ioannis G. Papagiannis, MD serves as a speaker on the Advisory Board for Horizon Therapeutics. The other author has no conflicts of interest to disclose.
